# Over-the-counter provision of near-vision spectacles: opportunities to grow

**Published:** 2026-03-12

**Authors:** Shadrack Muma, Kovin Naidoo, May Ho, Priya Morjaria

**Affiliations:** 1 Principal Investigator: OneSight EssilorLuxottica Foundation and Programme Coordinator: Let Our Children See, Kisumu, Kenya.; 2 Global Head: Advocacy and Partnerships: OneSight EssilorLuxottica Foundation, Durban, South Africa.; 3 Optometry and Primary Care Adviser: The Fred Hollows Foundation, Melbourne, Australia.; 4 Assistant Professor: London School of Hygiene & Tropical Medicine and Head of Global Programme Development: Peek Vision, UK.

**Eye care professionals should be at the forefront of strengthening over-the-counter provision so that we can scale up service provision for all, while improving access to specialist services for those who need them.**

**Figure:**
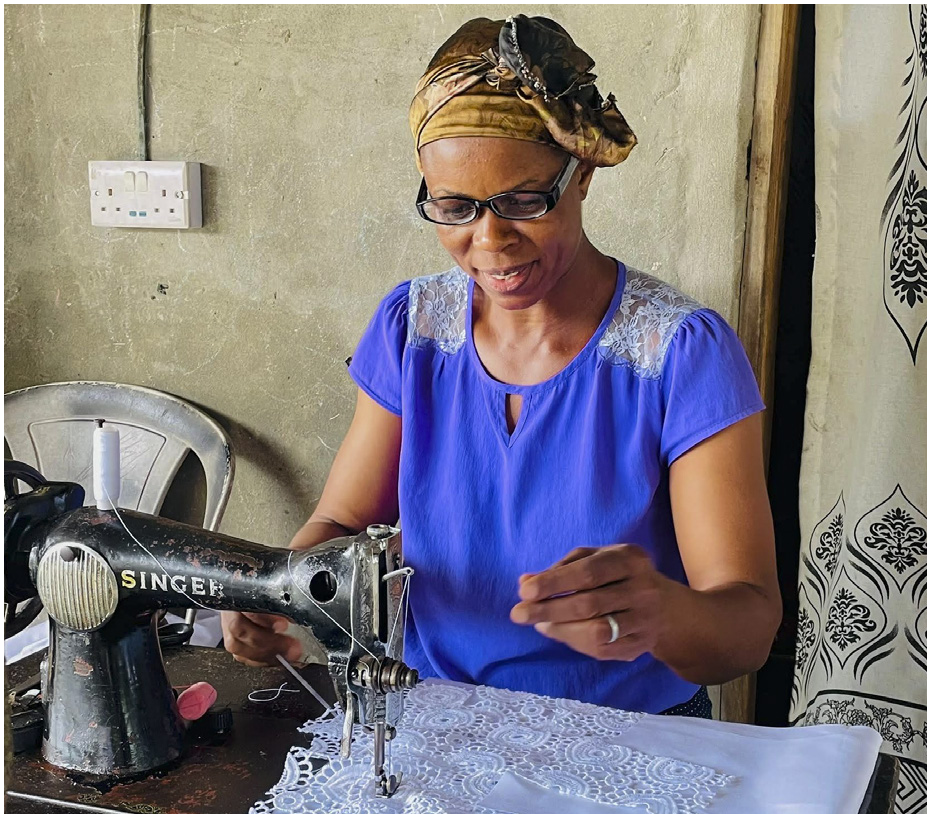
Near-vision spectacles improve lives and livelihoods. NIGERIA

© NESHP/CHAI CC BY-NC-SA 4.0

Presbyopia is an age-related decline in near vision caused by reduced accommodation of the eye. It affects hundreds of millions of people worldwide and is among the leading causes of near-vision impairment, contributing to an estimated annual productivity loss of USD 25 billion.^1^

Several studies have highlighted that ready-made near-vision spectacles - pre-made spectacles (often mass-produced) for presbyopia that have the same spherical power in each eye - can effectively provide correction for 44-60% of adults with near-vision impairment.^2^ They are considered low-risk medical devices and are designed for safe self-selection, with evidence showing that most users can choose the correct power. They are also inexpensive to manufacture and distribute, often costing just a fraction of the price of customised prescription spectacles.

As mentioned on p. 2, providing ready-made near-vision spectacles can increase productivity by around 22% and boost income by up to 33%. Yet, access to this simple, low-cost intervention remains limited, especially in low- and middle-income countries - where up to 86% of the population have presbyopia that remains uncorrected, compared to just 1% in high-income countries (Figure 1).^4^

Because the need is so great - and correction (using ready-made near-vision spectacles) is simple, safe, and effective for the vast majority of people - the World Health Organization (WHO) has recommended that presbyopia can also be addressed by non-eye care professionals (see article on p. 4 in this issue).

**Figure 1:**
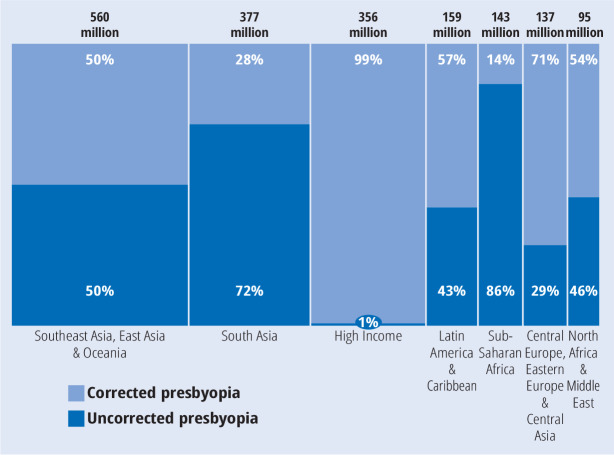
Number of people* estimated to have presbyopia by region (in millions), with percentages estimated to have corrected and uncorrected presbyopia.^3^

* 1.8 billion people are estimated to have presbyopia, of whom 826 million are uncorrected.^3^

This is good news, as there are not enough optometrists, opticians, or ophthalmologists to meet the overwhelming need, especially in low- and/or middle-income countries. For example, in sub-Saharan Africa, where 86% of presbyopia is uncorrected,^3^ there are only 2.5 ophthalmologists and 7.5 optometrists per million (WHO recommends a minimum of 4 ophthalmologists and 10 optometrists per million population). These are located mainly in urban areas, creating a disparity in access, particularly in rural areas.^4^ If the dispensing of near-vision spectacles were restricted to qualified eye health professionals only, most people with presbyopia would remain uncorrected, perpetuating avoidable vision impairment and productivity losses.

WHO recommends two complementary approaches to close the gap in access:

1. Providing ready-made near-vision spectacles via existing primary and community health care systems as part of an integrated, competency-based refractive error team (see article on pp. 6-7).
2. ‘Over-the-counter’ provision in places such as pharmacies, shops, and community-level outlets, which is discussed in this article.

## The case for over-the-counter provision

Making it possible to purchase ready-made near-vision spectacles over the counter - without the need for a prescription from an accredited eye care professional - is a practical, safe, and scalable way to close the gap in unaddressed need.

Over-the-counter provision has been tried and tested in most high-income countries: it removes unnecessary barriers to care, enabling hundreds of millions of people to access affordable near-vision correction quickly and safely. In low- and middle-income countries, where access to general eye care services is more limited, WHO notes the need for comprehensive eye care services in tandem with over-the-counter provision of near-vision spectacles. This ensures that people whose vision does not improve with ready-made near-vision spectacles can access the refractive and eye care services they need. Given the scale of the need, and the positive impact on productivity and quality of life, over-the-counter provision of near-vision spectacles should not be delayed.

In all countries, over-the-counter access offers opportunities to inform and educate members of the public about the importance of regular eye examinations and to direct them to other eye health services as needed.

## The benefits to optometry and ophthalmology providers

### Market growth

By acting as a local access point for initial eye health information and making referrals to specialist services as needed, over-the-counter provision can increase the number of people seeking eye care from optometrists or ophthalmologists, thereby supporting a growing eye health care system.

In regions where ready-made near-vision spectacles can be purchased without the need for a prescription from an accredited eye care professional, optometry and ophthalmology have continued to grow. Evidence from countries where ready-made near-vision spectacles are widely available shows that, once people adjust to wearing them, they often purchase additional pairs: one for work, one for home, or higher-quality customised spectacles later on.^5^ This creates sustained business for outlets and provides a pathway for patients to transition from over-the-counter solutions to professional eye care when needed.

### Reducing the burden on existing services

By shifting simple presbyopia correction away from clinical settings, eye care professionals are freed to concentrate on diagnosing and managing more complex refractive errors and eye diseases. This not only increases access for people living in remote settings, but also reduces the heavy dependence on hospitals and specialist eye clinics, which are often concentrated in urban areas and already overstretched.

By linking with local outlets, promoting local eye care services, and supporting the education of people working in such outlets, local practitioners can help to strengthen referral pathways and ensure that people receive the eye care they need (see panel).

### Demand generation for eye care and greater acceptance of spectacles

When households have easy access to ready-made near-vision spectacles, vision correction becomes normalised and less intimidating, provided vendors understand the need for awareness creation and referrals. Data collected by eye health organisations in India and sub-Saharan Africa (using Peek software) show that people with presbyopia who already own near-vision spectacles when they undergo vision screening are more likely to attend their referral appointments. This creates a virtuous cycle where entry-level access to spectacles helps to build awareness, reduces stigma, and ultimately drives demand for comprehensive eye health services.

Expanding access to ready-made near-vision spectacles aligns closely with the global agenda of achieving the WHO 2030 targets of effective coverage for refractive error by ensuring that essential, low-cost, high-quality interventions reach the people who need them most. Given the burden of presbyopia, ready-made near-vision spectacles are one of the simplest and most effective ways to address it. The over-the-counter approach does not diminish the role of optometrists and ophthalmologists; rather, it frees them to focus on complex and higher-risk cases, and it can be leveraged as an opportunity to increase health-seeking behaviour in the wider population - improving acceptance and enhancing the market for optometry and ophthalmology.

**Making the most of over-the-counter provision**

Over-the-counter provision can be enhanced in several ways, creating opportunities to raise public awareness of both presbyopia and other eye conditions and encourage more people to access other eye care services - thereby boosting the eye care sector. Eye care professionals should be at the forefront of these efforts, so that we can scale up service provision for all while improving access to specialist refractive error and eye health services for those who need it.

**NOTE:** Implementation of the suggestions below should not be prioritised in a way that will delay the availability of over-the-counter ready-made near-vision spectacles for presbyopia, which are safe and have a significant, positive impact on livelihoods and productivity.

**1. Training for non-eye care professionals**

To make the most of the opportunities offered by over-the-counter provision, some countries may wish to collaborate with eye care practitioners to provide non-eye care professionals with basic orientation or training on presbyopia, the selection of ready-made near-vision spectacles, and the importance of encouraging customers to seek eye care if they have additional needs or concerns. This 8-minute training video was created by RestoringVision: bit.ly/RVnvtraining for use in their programmes.

By educating customers on presbyopia, the safe use of near-vision spectacles, and the importance of seeking professional care when symptoms persist or other eye conditions are suspected, people working in over-the-counter providers can help bridge knowledge gaps at the community level. Local eye care professionals can also offer information and education sessions to outlets in their catchment area, thereby creating mutually supportive partnerships.

**2. Eye health messaging**

People working in over-the-counter outlets can be encouraged to help raise awareness of eye health, e.g., about the importance of regular eye examinations. This can be done either verbally or by displaying public health messages at the point of sale, such as posters encouraging customers to seek eye care if near-vision spectacles do not improve their sight or if they have additional needs or concerns (see examples in this issue).

Eye care professionals can partner with local outlets by providing public education messaging and even leaving their business card, so customers know where they can go to seek further help if needed.

**3. Quality standards**

Over-the-counter dispensing presents an opportunity for countries to implement quality standards for ready-made near-vision spectacles, as noted by WHO.
